# Diet Quality, Healthy Dietary Restrictions, and Adherence to the Mediterranean Diet in Food Deserts Among the Elderly in Spain

**DOI:** 10.3390/nu17020255

**Published:** 2025-01-11

**Authors:** Miriam Carmena del Viso, Ricardo Mora, David Navarrete-Villanueva, Isabel Iguacel

**Affiliations:** 1 Centro de Salud de Alcañiz, C. Andrés Vives, s/n, 44600 Alcañiz, Spain; mcarmena@salud.aragon.es; 2 Neonatal Intensive Care Unit (NICU), University Hospital Lozano Blesa, 50009 Zaragoza, Spain; ramora@salud.aragon.es; 3 EXER-GENUD (Growth, Exercise, Nutrition and Development), Faculty of Health, University of Zaragoza, 50009 Zaragoza, Spain; 4 Instituto Agroalimentario de Aragón (IA2), 50013 Zaragoza, Spain; iguacel@unizar.es; 5 Instituto de Investigación Sanitaria Aragón, 50009 Zaragoza, Spain; 6 NUTRI-GENUD (Growth, Exercise, Nutrition and Development), Faculty of Health Sciences, University of Zaragoza, 50009 Zaragoza, Spain; 7 Centro de Investigación Biomédica en Red de Fisiopatología de la Obesidad y Nutrición (CIBEROBN), Instituto de Salud Carlos III, 28029 Madrid, Spain

**Keywords:** food deserts, diet quality, food access, Mediterranean diet, COVID-19

## Abstract

Background/Objectives: Food deserts are areas characterized by limited access to affordable and healthy food, often due to significant distances from supermarkets—exceeding 1.6 km in urban areas and 16 km in rural settings. These spatial limitations exacerbate health and socioeconomic disparities. This study aimed to assess diet quality and explore the barriers influencing dietary behaviors among individuals aged 60 and older residing in food deserts in Aragón, Spain. Methods: An exploratory qualitative study using a phenomenological approach was conducted, complemented by descriptive analyses of sociodemographic data and adherence to the Mediterranean diet, assessed through the PREDIMED questionnaire. Data were collected through in-depth interviews and a focus group with residents of food deserts. Results: Half of the participants showed high adherence to the Mediterranean diet, while the other half had moderate adherence. Despite the lack of access to supermarkets and higher food costs, many participants perceived their diet quality positively. Challenges, such as long distances to stores, limited public transport, and economic constraints, were mitigated through strategies like home gardening, hunting, and traditional cooking, which often reduced reliance on processed foods. However, participants also reported increased alcohol consumption as a means of socializing. Conclusions: Diet quality in Aragón’s food deserts reflects a balance between significant access barriers and community-driven resources. While participants leveraged local strategies to maintain diet quality, systemic issues remain. Public policies should focus on improving transportation, enhancing local food systems, and fostering community-based initiatives to reduce inequalities in food access and promote healthier dietary habits in rural areas.

## 1. Introduction

The concept of food deserts, first introduced in Scotland during the 1990s, has since developed into a pivotal framework for understanding disparities in food access and diet quality. According to the United States Department of Agriculture (USDA), food deserts are areas where individuals face limited access to a variety of healthy and affordable foods, thereby restricting opportunities to maintain a balanced diet [1,2].

This limitation is often physical, defined by significant distances to supermarkets—over 1.6 km in urban areas or 16 km in rural areas [1,3]. Such geographic challenges exacerbate inequalities in transportation availability, shopping time, store variety, and ease of food acquisition [4].

In these areas, restricted access to fresh, healthy, and affordable food sources poses substantial barriers to achieving a balanced diet [3,5]. Studies have consistently linked these conditions to poorer diet quality and an elevated prevalence of health issues, including obesity, diabetes, or malnutrition [6,7,8].

To address disparities in food access, initiatives like mobile markets have emerged, offering fresh produce and other staples tailored to community needs. However, these efforts are often hindered by higher prices and limited product variety, reducing their effectiveness [9,10]. Additionally, access to private vehicles is critical in rural areas, where public transportation options are often lacking [1,11].

In Aragón, Spain, the prevalence of food deserts is shaped by the region’s low population density and significant geographic dispersion, a legacy of rural-to-urban migration. Population densities vary dramatically across Aragón’s provinces: Teruel (9.1 inhabitants/km^2^), Huesca (14.4 inhabitants/km^2^), and Zaragoza (55.9 inhabitants/km^2^) [12,13,14].

During the COVID-19 pandemic, supply chain disruptions exacerbated food access issues in food deserts. Strict lockdown measures further strained resources, although studies indicate these impacts were more severe in urban areas [15,16,17,18].

The link between diet quality and chronic non-communicable diseases highlights the need to address food access issues in food deserts. The Mediterranean diet, recognized for its health benefits, is associated with reduced risk of chronic diseases and longer life expectancy [19,20,21,22]. However, adherence to this dietary pattern remains low in Spain, with only 14.5% of the population reporting high adherence [23].

This study focuses on describing diet quality and the barriers to food access in Aragón’s food deserts. It explores factors such as home gardening, social support networks, and transportation to provide insights into dietary behaviors and challenges faced by this population in food deserts.

## 2. Materials and Methods

A qualitative exploratory phenomenological study was conducted and complemented by a descriptive analysis of sociodemographic variables and adherence to the Mediterranean diet from the Prevención con Dieta Mediterránea (PREDIMED) adherence questionnaire.

### 2.1. Study Design and Setting

In-depth semi-structured interviews were conducted with four individuals aged 60 and over residing in areas considered food deserts in the province of Teruel, specifically Ródenas and Torre de las Arcas. Additionally, a focus group consisting of six participants was conducted in Artieda in the province of Zaragoza. Both the interviews and the focus group were carried out between December 2022 and January 2023.

Field research was conducted in three rural regions: Sierra de Albarracín and Cuencas Mineras in Teruel and La Jacetania in Zaragoza. These locations are characterized by low population densities and limited access to fresh food:

Ródenas, with a population of 62 inhabitants (34 men and 28 women) [24], has a population density of 1.4 inhabitants/km^2^ (data from IAEST, 2022) [25]. Residents primarily work in agriculture and livestock farming. The nearest grocery store is in Bronchales, 22.7 km away (21 min by car).

Torre de las Arcas, with a population of 24 inhabitants (14 men and 10 women) [24], has a population density of 0.6 inhabitants/km^2^ (IAEST, 2022) [25]. Residents engage mainly in agriculture and livestock farming. The nearest grocery store is in Obón, 16 km away (36 min by car).

Artieda, with a population of 82 inhabitants (47 men and 35 women) [24], has a population density of 6 inhabitants/km^2^ (IAEST, 2022) [25]. Residents focus on agriculture, livestock farming, and rural tourism. The nearest grocery store is 20 km away.

The sample in this study had to meet the following inclusion criteria: being over 60 years old, residing in food deserts of Aragón (Spain), and being able to understand and consent to this study’s objectives. Weekend-only residents were excluded. These criteria applied to both the participants in the in-depth semi-structured interviews and those involved in the focus groups.

### 2.2. Variables and Measurement Methods

Before initiating this study, an in-depth analysis of food deserts in Aragón was conducted. To achieve this, we contacted local municipalities and health centers in the region to obtain detailed information regarding the presence of food stores and mobile markets. Towns and villages lacking physical stores or requiring a minimum 16 km drive to access fresh food were classified as food deserts. Food desert identification was conducted by listing all towns and villages in Aragón and documenting their access to grocery stores or supermarkets offering fresh produce. Data were gathered through Google Maps, the rural multiservice website, and direct communication with local authorities, nurses, and residents. This approach was necessary as such information was often unavailable through online resources like Google Maps or other internet platforms.

Once all the data about food deserts were consolidated into our database, we reached out to the mayors of the areas identified as having significant accessibility challenges. During these meetings, this project was explained in detail, including its objectives, the methodology, and the criteria for participant selection.

The mayors of the most affected areas provided information about residents who met the inclusion criteria (over 60 years old, residing in food deserts, and capable of understanding and consenting to this study). These individuals were then contacted by their respective mayors, who introduced this study and encouraged participation. Following this process, all individuals contacted agreed to participate in this study.

This tailored approach ensured the identification and inclusion of participants most representative of this study’s focus, leveraging local knowledge and community engagement.

Individual interviews were conducted in Ródenas and Torre de las Arcas by two research team members (D.N.V. and R.M.R.) in participants’ homes. The average duration of each interview was approximately 25 min., and the focus group in Artieda, consisting of six participants, was moderated by two researchers (I.I.A. and R.M.R.) and lasted 50 min. Conversations were recorded for later transcription and analysis. One team member acted as the moderator, guiding the conversation, while the other observed and took notes on significant interventions and interactions between participants.

Participants from Torre de las Arcas and Ródenas were recruited through the municipalities and local nursing professionals. In Artieda, recruitment was facilitated through personal connections with a team member. Recruitment materials, including project flyers, were distributed via local WhatsApp groups and municipal noticeboards.

Prior to interviews, participants received informational materials on healthy eating based on guidelines from the Harvard School of Public Health [26] and the Spanish Agency for Food Safety and Nutrition (AESAN) [27] through a document designed by the research team. Participants also completed a questionnaire gathering basic sociodemographic data and information on their social support networks. Additionally, participants completed the PREDIMED Mediterranean Diet Adherence Questionnaire, validated in Spanish by Schroder et al. in 2011 [28,29].

Study questions focused on purchasing habits, preparation, and consumption practices. The complete question guide is available in Table 1.

### 2.3. Data Analysis

Quantitative data from the descriptive analysis were tabulated and coded into two-dimensional tables using Microsoft 365 Excel. Univariate analysis was performed using the Jamovi software (v.1.6.23). For qualitative variables, means, standard deviations, and frequency distributions were calculated. For quantitative variables, central tendency measures were calculated.

Qualitative data analysis aimed to create thematic categories from the data, followed by the generation of concepts or theories. Transcriptions were imported into qualitative analysis software (ATLAS.ti 25) [30]. Data analysis followed the steps described by Braun and Clarke [31]:

- Familiarization and transcription: reading and rereading data, noting initial ideas, and transcribing data;
- Coding: assigning codes to relevant data systematically, forming small data groups;
- Categorization: classifying codes into potential themes and gathering relevant data for each potential theme;
- Definition of categories: identifying categories and subcategories that make up the data set;
- Review and interrelation between categories: relationships between categories and subcategories were analyzed to establish intrinsic connections. This step resulted in the development of a conceptual map;
- Final report: the findings were documented, linking the analysis to the study’s objectives and presenting conclusions based on the results.

The categorization process was followed by the graphical representation of the data collected in this study through a conceptual map. This map illustrated the thematic areas identified in this study and highlighted the interrelations among them, providing a clear visual summary of the concepts explored.

### 2.4. Validity and Rigor in Study Content

To ensure the quality and rigor of the data, reliability criteria for qualitative research, as established by Lincoln and Guba, were applied (Table 2) [32].

Triangulation strategies were used among different study participants, and the confirmability of results was guaranteed by including direct participant quotes. Additionally, to improve the quality, exhaustiveness, and credibility of the communication of results, the COREQ guidelines for qualitative research reporting were followed [33]. Before disseminating the results, they were sent to study participants for validation.

### 2.5. Ethical Considerations

This study adhered to the ethical principles for research involving human participants, as outlined in the Declaration of Helsinki (1964), the Belmont Report (1979), and Spanish regulations on biomedical research (Law 14/2007). This study received approval from the Aragón Ethics Committee (CEICA), with a favorable opinion issued in December 2022 (PL22/495).

It was also conducted following good clinical practice standards, ethical principles, and the aspects outlined in the Spanish Organic Law 3/2018 on the Protection of Personal Data and Digital Rights. The data collected were processed and stored in a lawful, fair, and transparent manner, guaranteeing anonymity and confidentiality.

## 3. Results

### 3.1. Description of Participants

A total of 10 participants met the inclusion criteria, signed the informed consent, and completed the questionnaires.

Participants’ ages ranged from 62 to 92 years, with a mean age of 72.6 years (standard deviation, SD = 10.2) and a median of 69 years. Most of the participants were women (70%), and 40% had a secondary education or lower. All participants were Spanish nationals, with 60% residing in the province of Zaragoza and 40% in the province of Teruel.

Regarding social support networks, only 20% of participants lived alone, while the remaining 80% reported having some form of cohabitation. Moreover, 80% of participants stated they could rely on three or more people in times of real need. Furthermore, 90% of participants had access to transportation (private car) to go to the nearest grocery store.

The sociodemographic results are shown in Table 3.

The results from the PREDIMED questionnaire, measuring the adherence to the Mediterranean diet, were as follows:

The minimum score obtained in the questionnaire was 6 points, with a mean score of 9.30 and a median of 9.50 points. Half of the participants had high adherence to the Mediterranean diet, and the other half had moderate adherence, with no participants exhibiting low adherence.

Regarding the responses to each item in the PREDIMED questionnaire, 100% of participants used olive oil as the main fat for cooking, drank fewer than one sugary beverage per day, and consumed fewer than seven glasses of wine per week. Moreover, 90% consumed more than one serving of red meat, hamburgers, sausages, or cold cuts per day, and fewer than one serving of butter, margarine, or cream per day. Additionally, 80% consumed two or more servings of vegetables per day and fewer than three servings of legumes per week. Furthermore, 70% consumed two or more servings per week of cooked vegetables, pasta, rice, or other dishes with sautéed vegetables, three or more servings of nuts per week, and four or more tablespoons of olive oil per day, and preferred chicken, turkey, or rabbit meat over beef, pork, hamburgers, or sausages. In addition, 60% consumed three or more pieces of fruit per day and fewer than two servings of industrial bakery products per week. Lastly, 50% consumed fewer than three servings of fish/seafood per week. These results can be seen in Table 4.

### 3.2. Qualitative Data Analysis

Through the coding process, four main themes emerged from the semi-structured interviews and focus group discussions. These themes were the following: (A) Perception of Diet Quality; (B) Barriers to Accessing Healthy Food; (C) Available Resources for Food Provision; and (D) Food Provision During the COVID-19 Pandemic.

#### 3.2.1. Perception of Diet Quality

All participants stated that they believed their diet was healthier compared to people living in the city who have better access to food.

- [200]: “*I think people in the city eat less healthy, for sure*.”

Several reasons were provided by the participants to explain why they believed they had a good-quality diet:

1. They ate home-cooked meals, avoiding processed supermarket food.

- [101]: “*Living in the village makes us eat better because we go for natural and homemade food. And second, all the processed stuff doesn’t get eaten here*.”

2. They had access to fresh, local food from practices such as home gardens, hunting, and raising animals. They also preserved meat through home slaughter.

- [103]: “*Hunting? Well, we also eat game here. At home, for example, we sometimes eat wild boar, roe deer, and quails. There are hunters at home, it’s not every day. But sometimes you eat it*.”

3. They bought fresh, high-quality products at mobile markets.

- [101]: “*Yes, this guy who comes on Tuesdays brings very good quality, very good products, like fresh vegetables.*”

Despite claiming to have a good-quality diet, participants acknowledged not strictly following healthy eating habits.

- [104]: “*We try to eat as naturally as possible, but I like a chocolate, I can’t resist, and if there’s a bowl of chips with glutamate, it’s so good. But well, I eat, I don’t overdo it*.”

Unhealthy habits were also identified, such as the lack of consumption of whole-grain bread and even harmful behaviors, like alcohol consumption.

1. Most participants reported consuming white bread from their village, perceiving it as superior in quality and consistency compared to whole-grain bread. While they acknowledged the availability of whole-grain bread, they noted that obtaining it was more challenging.

- [102]: “*In the village, we eat more white bread than whole-grain. [My son’s name] and I tried whole-grain bread for a while, but since no one likes it at home, I had to eat it alone*.”

2. Alcohol consumption was also noted during social gatherings, typically at the village bar, as a regular way of socializing.

- [201]: “*Well, I don’t know. I drink beer. For example, if I come at noon, I drink one, sometimes two, sometimes three. Then, in the afternoon, I try to go to the bar late, around 20:15 to 20:20. Some are watching football, others chatting*.”

#### 3.2.2. Barriers to Accessing Healthy Food

Physical, economic, and social barriers to accessing healthy food were identified, which led to changes in shopping habits. For example, participants reported the long distances they had to travel to the nearest store, the time spent shopping, and the frequency of shopping trips.

1. Physical Barriers

Within this group, participants mentioned the closure of food stores, the cessation of mobile market services, the distance to the nearest food store, and problems with public transport.

- [301]: “*There used to be a shop in the square that brought everything. Meat, fruit, and all kinds of things. But that closed a long time ago, and now we only have the services we have*.”
- [301]: “*Here I buy fruit and I used to buy meat, but now I don’t. Just last week, they closed the delivery truck*.”

Participants reported having to travel long distances to buy food, often visiting more than one store to complete their shopping for fresh produce, and going less frequently. The public transport system was also cited as inefficient.

- [102]: “*We travel to Jaca, Sangüesa, or Pamplona [nearby larger town], which amounts to nearly a 100-km round trip*.”
- [201]: “*I do the big shopping once a month, and for that, I go to Teruel [nearby larger town]*.”
- [201]: “*I don’t know if the bus is coming now, I have no idea. But I think not. To go to Teruel, they took us to Monreal [nearby larger village], and from Monreal, you had to take another bus to Teruel. But for shopping, you have to go with your own car*.”

2. Socio-Cultural Barriers

Participants mentioned a decline in traditional food practices for self-sufficiency, such as home gardening, raising animals, and hunting, as well as a reduction in traditional meat preservation practices.

- [200]: “*Before, my aunt used to plant things. But she moved to Zaragoza, now she’s in a retirement home… My aunts, back in my father’s time, they used to bring stuff, but now nothing. And my brother doesn’t garden anymore because he can’t. Before, we didn’t buy anything, now we buy everything*.”

3. Economic Barriers

Participants noted that products at mobile markets were priced higher, and to obtain lower-priced goods, they had to travel long distances.

- [301]: “*The Ortega [surname of the person who brought fresh produce in a van] also charged more. But, of course, they have to travel. It was more expensive, more expensive than in Teruel, for example, but that’s how it was*.”

#### 3.2.3. Available Resources for Food Provision

Individual, community, and political resources were identified in the study villages to address the challenges of accessing food.

1. Individual Resources

Most participants reported using their car to travel to stores for food shopping.

- [301]: “*In my case, I go to Teruel. I’m self-employed, I take my car, go to Teruel, and buy at the Mercadona [a well-known supermarket in Spain], there*.”

Most participants mentioned having their own garden, and a few raised animals or hunted to provide themselves with fresh and healthy food. Additionally, they preserved meat through home slaughter.

- [104]: “*I don’t eat supermarket chicken because it’s soggy when you see it... I eat chicken from home*.”

2. Community Resources

In all three study villages, participants described a strong sense of community, expressing that they had an extensive support network. For instance, participants without gardens or those not involved in raising animals or hunting reported receiving fresh food from family members, friends, or neighbors who practiced these food-related activities. They also exchanged favors, such as transporting someone for shopping or purchasing items on their behalf.

- [103]: “*I don’t have a garden, but I eat from my brothers’ garden*.”

Mobile markets exist in the study villages, providing fresh food and adapting their products to the food needs of the residents.

- [301]: “*The Ortega [surname of the person who brought fresh produce in a van], if we asked for something for the elderly, he’d bring it. He had a store, so if we needed cleaning products, cookies, anything, he’d bring it*.”

One participant mentioned that public transportation services were very useful and important for food access, as an alternative to using a private car.

- [201]: “*The region has a service that started a year and a half ago. A small bus comes on Thursdays in the morning, picks people up from Torre de las Arcas, Castell, Palomar, Escucha, and the surrounding areas, and takes us to Montalbán [a larger village with a food store]. In my car, I would have gone more comfortably, but these public services are very important, though they arrived late*.”

3. Political Resources

In Artieda, there is a project called “Envejece en tu pueblo” (“Aging in Your Village”), where the town hall subsidizes midday meals for registered retirees, which are served in the local bar. They also set up a WhatsApp consumption group where local food products for sale are shared.

- [104]: “*The elderly meals are subsidized by the town hall*.”
- [101]: “*Here there’s an ‘Envejece’ project that includes four villages: Artieda, Salvatierra, Sangüesa, and Mianos. They do many things together for older adults, like the consumption group, where we get information on local food products for sale*.”

#### 3.2.4. Food Provision During the COVID-19 Pandemic

During the COVID-19 pandemic, mobile markets maintained the food supply and provided more personalized services while adhering to safety measures.

- [201]: “*The guy from the Palomar store brought what was needed, the vegetable vendor came too, and we bought. The baker also came, all maintaining distance*.”

The community support network in all three study villages intensified. For example, if someone had to travel, they would arrange for someone to buy food on their behalf.

- [101]: “*We would order things, and [the person’s name], a girl involved in the ‘Aging’ project, would buy everything for us and bring it*.”

Additionally, community initiatives like creating a village WhatsApp group were implemented for facilitating shopping.

- [101]: “*We had a WhatsApp group, and there was a person who would buy the things and bring them to our house, a person from the village*.”

In cases where family networks were possible, they continued to provide food during the pandemic.

- [301]: “*In my case, it was easy because I have my daughters in Teruel, my son-in-law has a pig farm here, and he had to come every day. If something was missing, he would bring it*.”

## 4. Discussion

This study contributes to the limited research on the perceptions of people living in rural food deserts in the Autonomous Community of Aragón (Spain) regarding the quality of their diet. From the analysis of data provided by participants through in-depth semi-structured interviews and a focus group, food resources and barriers to accessing healthy food were identified, along with the impact of COVID-19 on food supply in the studied areas.

In the present study, residents in rural food deserts of Aragón reported having good diet quality. In fact, participants in our study showed moderate-to-high adherence to the Mediterranean diet, which is associated with better nutritional quality (Ejeda and Rodrigo, 2021) [34]. According to Baladia et al. (2022) [23], adherence to the Mediterranean diet increases with age, with 20.2% of people over 65 years old showing high adherence to this dietary model. These results are consistent with those of our study sample, where no low adherence was observed, and all participants fell within the moderate-to-high adherence range. However, these results differ from studies that associate living in food deserts with a higher risk of poorer diet quality [6,35]. It should be noted that both studies were conducted in the United States, where the Mediterranean diet is not followed.

However, residents in food deserts in Aragón face physical, socio-cultural, and economic barriers that hinder access to healthy food and lead to changes in shopping habits. For example, the closure of food stores and mobile markets prevents access to fresh, quality, and affordable food [5]. Yet, studies show that a healthy diet is not only dependent on the presence of food stores offering healthy foods. According to Stern et al. (2016) [36] and Vaughan et al. (2017) [37], shopping at supermarkets is not always associated with purchasing healthy foods. Allcott et al. (2019) [38] argued that building a grocery store in a food desert had minimal effects on diet and is not a solution to improve nutritional status. Therefore, it is essential to consider other resources available in rural food deserts for food provision and maintaining a healthy diet.

Another barrier to good diet quality is the frequency of shopping trips. According to Kim et al. (2020) [4], longer shopping cycles result in buying more food than necessary, reducing its utility and increasing problems related to freshness, quality, and food safety. Rebouillat et al. (2020) [11] noted that people living closer to markets shop more frequently and buy less in quantity.

One of the most used resources for accessing food is private car ownership, with 90% of participants having a car in the household. Studies have shown higher car ownership in rural areas [1,37]. The villages included in this study have deficient public transport, making car ownership crucial for accessing healthy food and affecting shopping habits. Previous studies have associated access to a vehicle or public transport with greater availability of healthy and affordable food [39,40]. Political interventions aimed at increasing access to healthy food should consider transportation availability, whether public or private.

The social and cultural environment also influences food access; 80% of participants stated that they could rely on three or more people in times of real need. Smith and Morton (2009) [41] suggested that the presence of strong family and community social support networks is more prevalent in food deserts, where sharing food with family, friends, and community members is common. For example, sharing fresh food from traditional self-sufficiency practices such as gardening [10].

When accessibility, material resources, and social support networks are lacking, mobile markets play a crucial role in reducing barriers to healthy food access by bringing fresh, quality products closer to consumers [11]. However, this reduction may be affected by higher product prices. Participants in this study reported traveling to more distant stores to find lower prices. According to Robinson et al. (2016) [9], mobile markets adapt their products to customer needs and sell them at a higher price, which aligns with our findings. Miller et al. (2016) [42] noted that residents of Alderson (a food desert in West Virginia, USA) traveled over 17.7 km to reach the nearest grocery store and save money.

COVID-19 lockdown measures influenced access to healthy foods due to disruptions in the food supply and their associated effects on prices [43,44]. However, residents of Aragón’s food deserts reported minimal negative effects of the pandemic on food access for their diets, even during the strictest lockdowns. Mobile markets continued to supply food normally, and existing resources (community and family support) were strengthened, while new ones (WhatsApp groups) emerged, enabling good food access while complying with the imposed safety regulations.

The phenomenon of food deserts and their impact on older adults is not limited to specific regions; it is a global issue with significant disparities between countries and income levels. In high-income countries, food deserts are often concentrated in rural areas and socioeconomically disadvantaged urban neighborhoods, where access to fresh, nutritious food is restricted by geographic and economic barriers [45]. Conversely, in low- and middle-income countries, the problem is exacerbated by inadequate infrastructure, insufficient market availability, and systemic poverty, which limit food diversity and accessibility [46]. These disparities underline the need for context-specific interventions to address the unique challenges faced by older adults in different regions. Understanding these global patterns is essential for developing policies and programs that promote equitable access to healthy foods, thereby reducing the nutritional and health disparities associated with food deserts [47].

The findings of this study highlight the critical impact of food deserts on the nutrition and overall health of older adults. Poor access to nutritious food not only exacerbates existing health conditions but also increases the risk of developing new ones, particularly cardiovascular diseases [48]. In fragile individuals, inadequate nutrition can lead to hypertension, dyslipidemia, and other cardiovascular risk factors, which further contribute to morbidity and mortality [49]. These outcomes underline the importance of addressing food accessibility as a public health priority, particularly in regions with a high prevalence of food deserts [50]. Interventions that improve access to fresh and healthy foods, such as mobile markets or community-supported agriculture programs, could play a crucial role in mitigating these risks and improving the quality of life for vulnerable populations [51].

### Strengths and Limitations of This Study

Several studies have highlighted the value of considering the individual as the unit of analysis. Research on how people perceive their access to food can influence diet quality just as much as objective measures [52]. Despite this, no qualitative studies have analyzed the subjective perceptions of people living in rural food deserts in Spain. Therefore, this research represents an important step toward developing appropriate strategies to minimize diet inequalities between food deserts and other areas. By exploring the lived experiences of residents in food deserts in Aragón, this study aims to provide a deeper understanding of their complexities, increase knowledge, foster future research, and address food inequalities [53].

However, this study also has limitations that should be acknowledged. Although the initial goal was to conduct this study across all of Aragón’s food deserts, logistical challenges and geographical dispersion led to a focus on the province of Teruel, which has the highest number of food deserts in the Aragón region [54]. Nevertheless, one food desert from the province of Zaragoza was included to provide a broader perspective. This study can serve as a pilot for future research encompassing food deserts throughout the entire Aragón region.

Regarding the sample size, qualitative studies almost always use small, non-random samples. This does not imply a lack of concern for the quality of the sample; rather, qualitative research applies purposive sampling to select participants who can provide rich and meaningful insights into the phenomenon under study [55]. The findings reflect the experiences and perspectives of a specific group of individuals and are not intended to be statistically representative.

One frequently raised limitation of qualitative approaches is the question of the representativeness of results. It should be understood that qualitative research does not aim to produce findings that are statistically generalizable beyond the study’s context. Instead, its primary objective is to gain an in-depth understanding of a phenomenon within a specific context, prioritizing depth over breadth. The findings of this study are context-dependent and reflect the lived experiences of older adults in selected rural locations in Aragón, which may differ from those in other food deserts or among different demographic groups [56].

Furthermore, while this study considered broader contextual factors such as socioeconomic conditions and health status, these variables were not systematically collected as part of the inclusion criteria. As such, the current results are not generalizable to other geographical areas, time periods, or demographic characteristics. Different findings might have emerged had this study been conducted with a different population or in a different territory [57].

Future research could address these limitations by including larger and more diverse samples, incorporating additional demographic variables, and expanding the geographic scope to better understand how various factors influence food access and dietary behaviors in food deserts [58].

## 5. Conclusions

This research suggests the need to go beyond the concept of a food desert as a simplified way of understanding food access. The distance to the nearest food store cannot define whether a village is or is not a food desert, according to the strict meaning of the term. The rural areas of food deserts included in this study possess a series of self-sufficiency resources and a wide socio-familial support network, which help mitigate barriers to healthy eating in these regions.

The diet quality of residents in food deserts in Aragón depends on a series of interrelated factors. The result of the interactions between available food resources and barriers to accessing healthy food define the acquisition and purchasing patterns in this population. Further research is needed to examine the association between these acquisition and purchasing patterns and dietary intake and health outcomes.

Public entities should promote public benefit programs, allocating part of the budget to reducing barriers to food access in Aragón’s food deserts, especially in areas lacking self-sufficiency resources, social support networks, or private transportation.

## Figures and Tables

**Table 1:** Interview and focus group question guide.

Interview and Focus Group Question Guide	Content
How do you follow the Mediterranean Diet’s guidelines?	Analysis of how participants apply the recommendations of the Mediterranean diet.
Problems and barriers to following the guidelines	Identification of barriers and their causes related to adherence to the guidelines.
Influence of living in this village on diet	Reflection on how living in the village affects eating habits.
Food shopping and acquisition	Locations where participants buy food and shopping frequency.
Means of transport used for shopping	Modes of transport employed to access shops or markets.
Support for food shopping or access to fresh food	Availability of help from others to obtain fresh food.
Commonly consumed foods	Types of food included in the participants’ daily diets.
Methods of food preparation	Common ways of preparing food.
People available for support in times of need	Number of people that participants can rely on during critical situations.
Availability of a home garden	Assessment of whether participants have their own garden.
Resources participants feel they lack	Identification of unmet needs in terms of food access.
Changes in the situation over recent years	Changes in diet or resources during the pandemic and lockdown.

**Table 2:** Quality and rigor criteria in qualitative research.

Criteria	Description
Credibility	Verification of results through immersion of researchers in the studied phenomenon, data triangulation, and peer review.
Transferability	Detailed description of study context, sampling methods, and participant characteristics, enabling replication of the study.
Reliability	Transparency in data collection, transcription, and analysis processes.
Confirmability	Analysis conducted following established scientific criteria, ensuring neutrality during data collection and interpretation.

**Table 3:** Sociodemographic characteristics of the study sample (N = 10).

	Characteristics	n	%
Age	60–69 years	5	50%
	over 69 years	5	50%
Gender	Male	3	30%
	Female	7	70%
	Other	0	0%
Country of Origin	Spain	1	100%
		0	0%
Province of Residence	Zaragoza	6	60%
	Teruel	4	40%
Education Level	Primary education	2	20%
	Secondary education	2	20%
	High school, 3rd BUP or COU, or medium level vocational training	3	30%
	Higher vocational training or higher-level FP	0	0%
	University, master’s, or doctorate	3	30%
Living Alone	Yes	2	20%
	No	8	80%
Support Network	None	0	0%
	One	1	10%
	Two	0	0%
	Three or more	8	80%
Use of Public Transport	Yes	9	90%
	No	1	10%

**Table 4:** Frequency of responses for each item in the PREDIMED Mediterranean Diet Adherence Questionnaire.

Items	Freq.	%
Item 1: Do you use olive oil as your primary cooking fat?	Yes	100%
Item 2: How many tablespoons of olive oil do you consume daily?	4 or more	70%
Item 3: How many servings of vegetables do you consume per day?	2 or more (including one raw or salad)	80%
Item 4: How many pieces of fruit do you consume per day?	3 or more	60%
Item 5: How many servings of red meat, hamburgers, sausages, or cold cuts do you consume per day?	Less than 1	90%
Item 6: How many servings of butter, margarine, or cream do you consume per day?	Less than 1	90%
Item 7: How many sugary or carbonated beverages do you consume per day?	Less than 1	100%
Item 8: Do you drink wine? How much do you consume per week?	Less than 7 glasses	100%
Item 9: How many servings of legumes do you consume per week?	Less than 3	80%
Item 10: How many servings of fish/seafood do you consume per week?	Less than 3	50%
Item 11: How often do you consume industrial bakery products like cookies, cakes, or pastries?	Less than 2 per week	60%
Item 12: How many times per week do you consume nuts?	3 or more	70%
Item 13: Do you prefer chicken, turkey, or rabbit over beef, pork, hamburgers, or sausages?	Yes	70%
Item 14: How often do you consume cooked vegetables, pasta, rice, or other dishes with sautéed vegetables?	2 or more per week	70%

## Data Availability

The data presented in this study are available on request from the corresponding author. The data are not publicly available due to ethical reasons.
